# Uterine Metastasis From Lobular Breast Carcinoma: A Case Report

**DOI:** 10.7759/cureus.68943

**Published:** 2024-09-08

**Authors:** Aniqa Faraz, Sydni Kowalczyk, Mark Hendrixson

**Affiliations:** 1 Internal Medicine, Cumberland Medical Center, Crossville, USA; 2 Oncology, Lincoln Memorial University DeBusk College of Osteopathic Medicine, Harrogate, USA; 3 Oncology, Cumberland Medical Center, Crossville, USA

**Keywords:** abnormal vaginal bleeding, breast cancer metastasis, cancer, endometrial metastasis, lobular cancer

## Abstract

There have been a few reports of extra pelvic tumors migrating into the female genital tract. Patients with postmenopausal vaginal bleeding are typically suspected of having primary uterine cancer. We present a case of lobular breast cancer that manifested about two years later as an atypical vaginal bleeding and was found to have endometrial and myometrial metastases. Pathological evaluation of endometrial and endocervical tissue samples revealed that breast cancer was the underlying cause.

## Introduction

Invasive lobular carcinoma (ILC), which accounts for 10%-15% of the total cases of breast cancer, is the second most common subtype after invasive ductal carcinoma (IDC). Breast cancer is the most common cancer among women worldwide [1]. An ILC is noted for its distinct histological features and a unique pattern of metastasis, including a propensity for spreading to unusual sites. Breast cancer tends to spread to the brain, liver, lung, and bone; on the other hand, metastasis to the uterus is extremely uncommon and presents major hurdles for both diagnosis and treatment [1]. 

Uterine metastases from breast cancer, particularly lobular carcinoma, are extremely rare; only a few cases have been reported in the literature. This infrequency may cause delays or misdiagnosis in certain cases, especially when patients present years after the original diagnosis of breast cancer with nonspecific gynecological symptoms such as irregular postmenopausal bleeding [2]. This case report describes a unique example of uterine metastasis from lobular breast cancer, highlighting the need for patients with a history of breast cancer and new gynecological symptoms to be closely monitored for metastatic disease. In order to improve outcomes through prompt identification and treatment, it is essential to document such cases to enhance our understanding of this uncommon metastatic pathway [3]. 

## Case presentation

A 65-year-old woman was sent to a gynecologist in 2021 for abnormal postmenopausal vaginal bleeding that began a few weeks earlier. Her medical history included breast cancer, hypertension, osteoarthritis, and anxiety. At 52, she had gone through menopause. After a pelvic exam, the cervix and uterus were found to be enlarged, and there was a tiny amount of bloody vaginal discharge.

The patient underwent a right breast lumpectomy in 2019 due to ILC of the right breast. Chemotherapy and radiation therapy were subsequently given. She received four cycles of doxorubicin 60 mg/m^2^ and cyclophosphamide 600 mg/m^2^ with pegfilgrastim support, followed by 12 weekly doses of Abraxane 80 mg/m^2^. After receiving Abraxane for 12 weeks, she started to have peripheral neuropathy at her fingertips and dizziness. She underwent Tamoxifen endocrine therapy. The patient had lobular stage IIA (pT2pN0(1+)) breast cancer with a 3.5 cm mass, 100% positive for the estrogen receptor and 50% positive for the progesterone receptor, and negative for human epidermal growth factor receptor 2. Her most recent annual mammogram and the Signatera test for circulating tumor DNA (ctDNA) came out negative during her yearly follow-ups.

**Figure 1:**
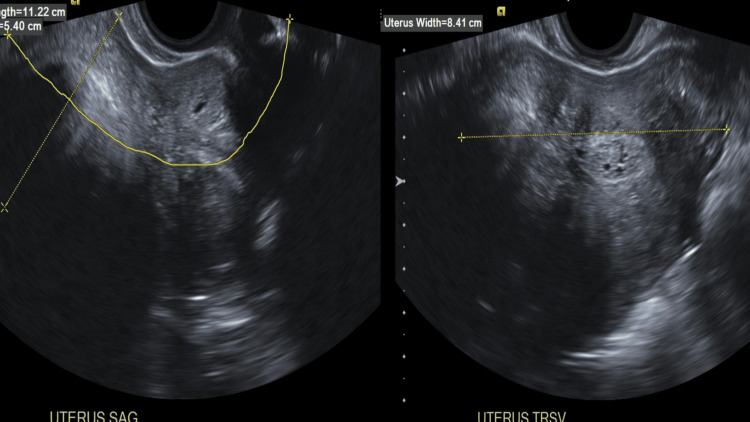
A transvaginal ultrasound (sagittal and transverse view) revealed an enlarged uterus measuring 11.2 x 5.4 x 8.4 cm with heterogeneous myometrial echotexture.

A diagnostic workup was initiated to detect possible causes of postmenopausal vaginal bleeding. Transvaginal ultrasound revealed an enlarged uterus that measured 11.2 x 5.4 x 8.4 cm and had a heterogeneous myometrial echotexture, as shown in Figure 1.

**Figure 2:**
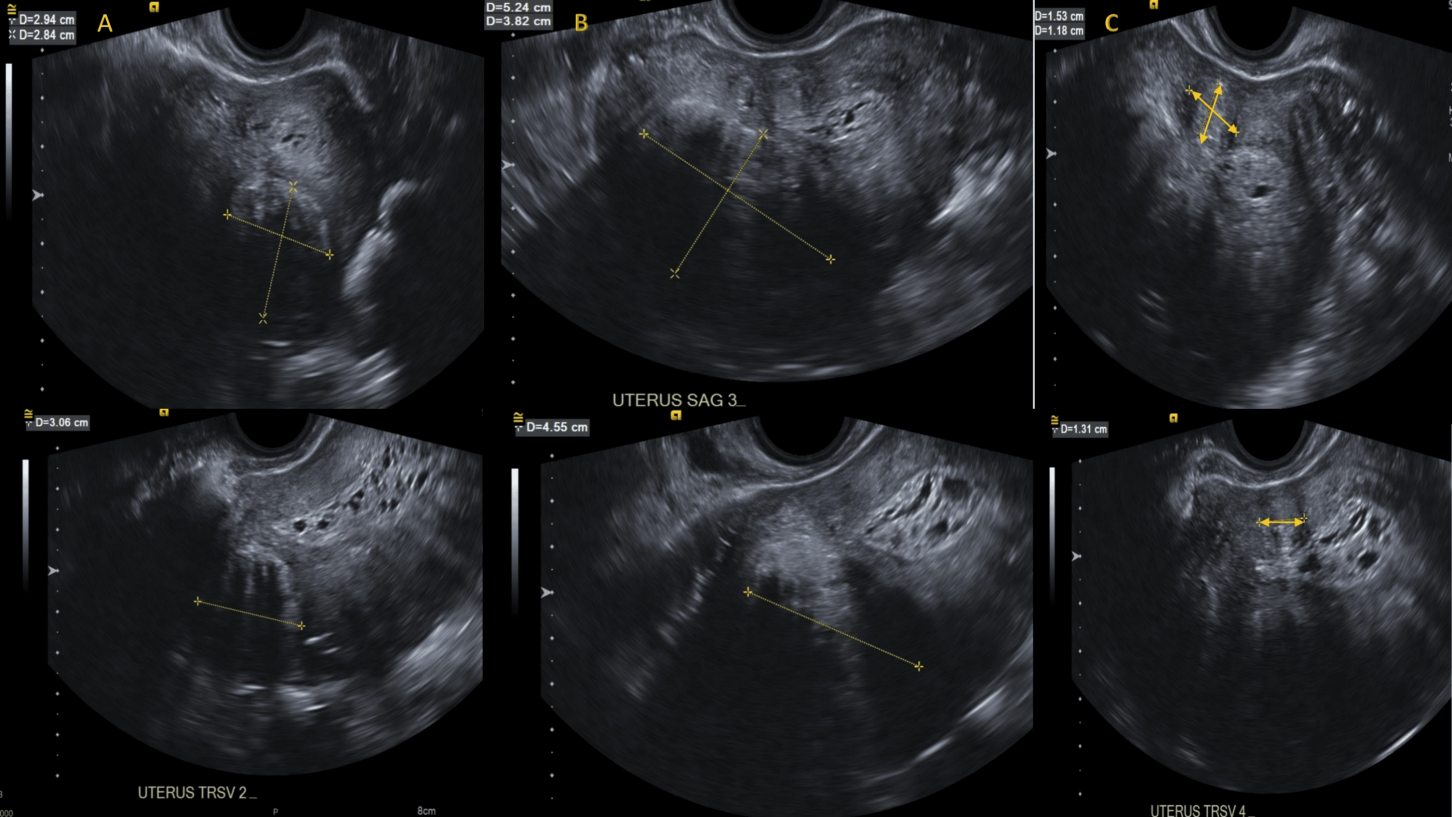
Several different-sized myometrial lumps located in various places on the uterus were visible on a transvaginal ultrasound.

There were several myometrial lumps inside the body that were consistent with leiomyomas. A hypoechoic mass measuring 2.9 x 2.8 x 3.1 cm was located in the anterior lower uterine section on the sagittal scan (Figure 2A). In addition, the posterior uterine fundus included a large, partially calcified mass measuring 5.2 × 3.8 x 4.6 cm, along with posterior acoustic shadowing (Figure 2B). In the right anterior uterine fundus, a 1.5 x 1.2 x 1.3 cm isoechoic mass with shadowing was found (Figure 2C).

**Figure 3:**
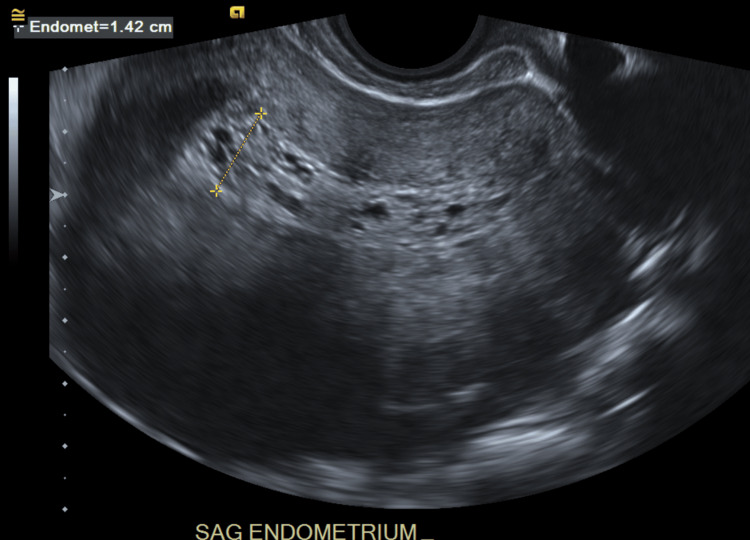
A transvaginal ultrasound sagittal view demonstrated endometrium thickening up to 14 mm.

The endometrial stripe was thickened, measuring up to 14 millimeters (mm), as demonstrated below in Figure 3.

**Figure 4:**
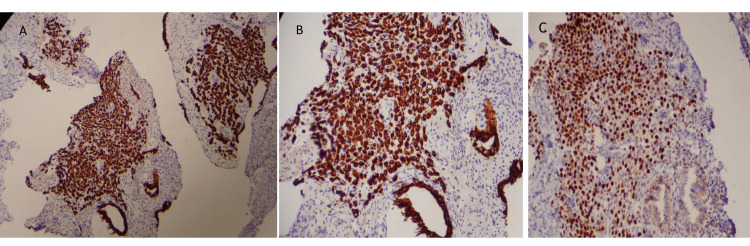
An IHC analysis of an endometrial specimen revealed sheets of positively stained tumor cells in the stroma, next to the endometrial glands, and beneath the surface endometrium for A) Pan-cytokeratin (AE1/3) at 100 times magnification; B) Pan-cytokeratin (AE1/3) at 200 times magnification; and C) GATA-3 nuclear IHC stain at 200x magnification. IHC: immunohistochemical

The abnormal postmenopausal bleeding, along with the ultrasound findings of an enlarged uterus and myometrial masses, prompted further investigation by hysteroscopy with biopsies, leading to the identification of metastasis. A hysteroscopy was performed, during which endometrial and endocervical tissue resections were carried out. Pathological analysis revealed metastatic non-small cell carcinoma of breast origin within the stroma of an otherwise benign polyp, exhibiting endometrial and endocervical features. The morphological characteristics were consistent with lobular carcinoma. No evidence of necrosis or angiolymphatic space invasion was observed. An immunohistochemical (IHC) profile was performed to help differentiate diagnosis, as in Figure 4. The stroma contained clusters of positivity for pan-cytokeratin (AE1/AE3) and cytokeratin 7, with a similar pattern of positivity for GATA-3. The stroma demonstrated scattered but not clustered positivity for CD68, a histiocytic marker. The stroma was entirely negative for cytokeratin 20. These strain results confirmed the presence of epithelial proliferation within the stroma, with an IHC straining pattern consistent with breast origin that would be metastatic in this location.

**Figure 5:**
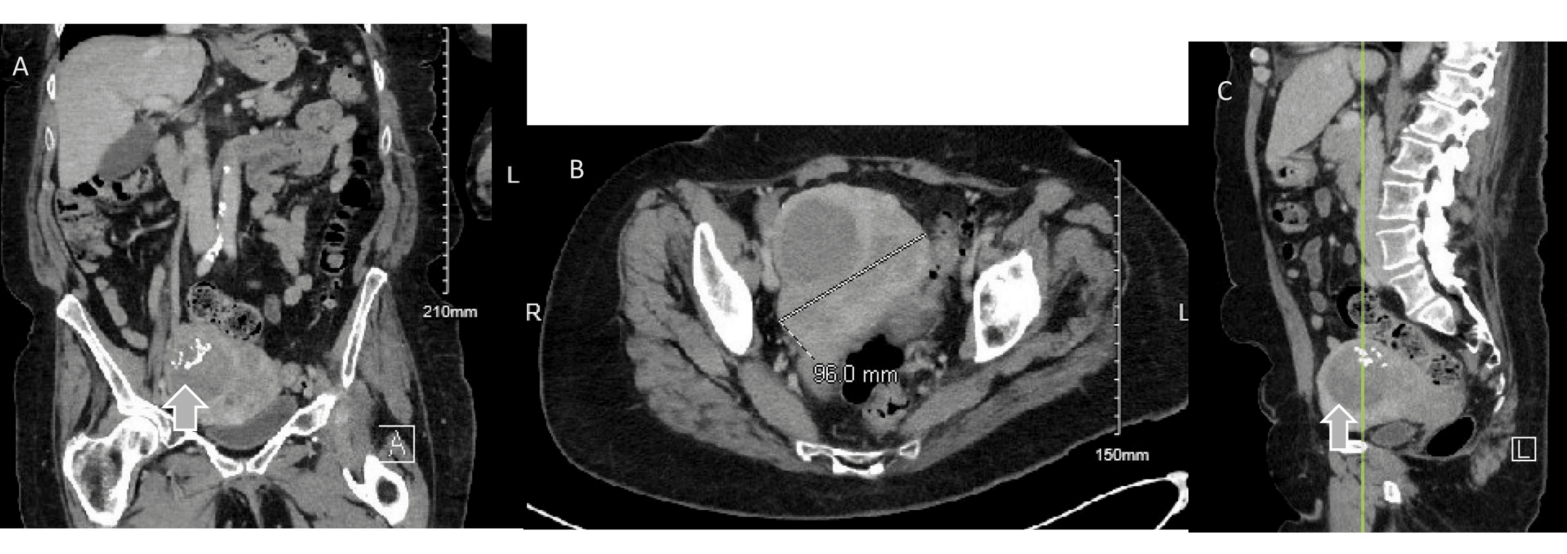
A CT scan of the chest, abdomen and pelvis (A) coronal, B) axial, and C) sagittal images) revealed the uterus to be significantly enlarged (measured at 11x9.6 cm) with multiple areas of cystic and calcific foci, presumably leiomyomatous enlargement.

After obtaining CT images of the chest, abdomen, and pelvis, the uterus was found to be considerably enlarged (measuring 11x9.6 cm), with many areas displaying cystic and calcific foci, likely indicative of leiomyomatous enlargement, as seen in Figure 5. There were additional subthreshold mediastinal and hilar lymph nodes seen.

After a multidisciplinary discussion, the patient received total abdominal hysterectomy (TAH) with bilateral salpingo-oophorectomy (BSO) and lymph node biopsies. The samples obtained from a polyp in the cervix, myometrium, bilateral fallopian tubes, and bilateral ovaries all contained metastatic carcinoma. Bilateral pelvic lymph nodes were all 4/4 positive for metastatic carcinoma. The morphological and immunophenotypic features were compatible with metastatic carcinoma from the breast as the primary. She then received a CDK-4/6 inhibitor (Ribociclib) and radiotherapy. The patient was not found to have any recurrence of lobular breast carcinoma during the onset of treatment for uterine cancer. 

## Discussion

The incidence of uterine metastasis from breast cancer is notably low, with metastatic lobular carcinoma to the uterus being sporadic. Lobular carcinoma, known for its unique ability to metastasize to unusual sites, presents a diagnostic challenge, often requiring a high index of suspicion and comprehensive diagnostic workup [3, 4]. One important pathogenic characteristic of ILCs is the lack of E-cadherin protein expression on the tumor cell membrane, which may be linked to the metastatic pathway of breast cancer. The E-cadherin-catenin complex malfunctions when E-cadherin is absent, which impacts cell adhesion. Metastatic tumor antigen (MTA) family proteins recruited by ZEB2 and G9A are regulated by GATA3, and its loss of expression or malfunction increases the metastasis of breast cancer. Inflammatory cytokines, such as interleukin-6 (IL-6), are crucial for the spread of breast cancer. In order to activate signal transducers and activators of transcription 3 (STAT3), IL-6 binds to the IL6R/gp130 complex and activates downstream of Janus kinase (JAK) signals. By controlling estrogen receptor α (ERα), which is necessary for breast cancer spread, IL-6/STAT3 signaling, in turn, encourages epithelial mesenchymal transition (EMT). The tumor microenvironment (TME) and immune cells could also play a role in the spread of breast cancer. Comparing IDC and ILC by clinical autopsy reveals that ILC spreads to gynecological organs more readily than IDC [5, 6].

Uterine metastasis from primary tumors outside the genital tract is uncommon. Most extragenital cases originate from gastrointestinal tract malignancies. The risk of primary uterine cancer increases in women who previously had breast cancer and were tamoxifen users [5-8]. Patients with breast cancer who are treated with tamoxifen have an incidence of endometrial cancer of 0.388%, which is probably more prevalent than uterine metastases [9].

The confirmation of endometrial metastases presents numerous diagnostic challenges. A few requirements must be met: (1) The metastatic tumor's histopathologic features must match those of the original neoplasm, with no evidence of concurrent primary neoplastic changes in the destination organ (uterus); (2) hormone-induced (tamoxifen-induced) endometrial neoplasm must be ruled out in known breast cancer patients; and (3) consistent IHC is required to identify the original tumor. In contrast to endometrial and breast carcinoma, which are CK7-positive and CK20-negative, GI cancers typically express CK20 and CDx2. In our case, the IHC results were completely compatible with ILC [10].

The management of uterine metastasis from breast cancer is not well defined, largely due to its rarity. In the treatment of breast cancer, metastasis is significant because it can serve as a target for therapy as well as a detectable marker. Her2, the metastasis rate, the metastasis site, and the presence or lack of hormone receptors all influence targeted treatment for metastatic breast cancer [6]. Treatment options may include systemic therapy aimed at primary breast cancer, localized treatment for uterine metastasis, or a combination of both [4]. Since the treatments and prognosis for a primary genital neoplasm and a metastatic breast tumor differ greatly, it is important to distinguish between the two. While surgical resection is an option for a primary uterine neoplasm, systemic chemotherapy is likely the better course of action for uterine metastasis [10]. 

In this case, despite the rarity of uterine metastasis, the patient's clinical presentation of abnormal postmenopausal bleeding warranted further investigation. This led to the discovery of uterine masses through transvaginal ultrasound and subsequent confirmation via hysteroscopy and histopathological examination. The decision to pursue TAH and BSO was made due to persistent vaginal bleeding, and it was followed by CDK-4/6 inhibitors and radiation therapy. 

## Conclusions

The present case report underscores the significance of doing a comprehensive evaluation of breast cancer survivors presenting with new gynecological symptoms by highlighting an unusual case of uterine metastases from lobular carcinoma. In addition to highlighting the vital necessity of surveillance beyond the initial treatment period, it also highlights the significance of a multidisciplinary approach for the best possible diagnosis and care. In our instance, the diagnosis of breast cancer metastases to the uterus was based on the ultrasound data previously described and hysteroscopy with biopsies that provided pathological confirmation. When breast cancer spreads to the uterus, there isn't a specific treatment available yet. In addition to postoperative radiation therapy, TAH and BSO can be utilized to stop the tumor's leftovers from spreading to other organs within the abdominal cavity.
